# Catalytic depolymerization of a typical lignite for improving tar yield by Co and Zn catalyst

**DOI:** 10.1038/s41598-017-14869-w

**Published:** 2017-10-31

**Authors:** Litong Liang, Juntian Huai, Qian Zhang, Jianwei Liu, Wei Huang, Zhonglin Zhang, Xiaogang Hao, Guoqing Guan

**Affiliations:** 1 Key Laboratory of Coal Science and Technology of Ministry of Education and Shanxi Province, Taiyuan University of Technology, Taiyuan, 030024 Shanxi China; 2 State Key Laboratory of Coal-based Low carbon Energy, ENN Group Co., Ltd, Langfang, 065001 China; 3 Department of Chemical Engineering, Taiyuan University of Technology, Taiyuan, 030024 Shanxi China; 4 North Japan Research Institute for Sustainable Energy, Hirosaki University, 2-1-3 Matsubara, Aomori, 030-0813 Japan

## Abstract

A novel process for improving tar yield through catalytic depolymerization of a typical lignite was put forward. The catalytic depolymerization of the lignite over CoCl_2_ and ZnCl_2_ catalyst in different concentrations were investigated by a Gray-King apparatus. The optimal catalyst concentrations of CoCl_2_ and ZnCl_2_ catalyst that could obtain the highest tar yield was achieved, by which the tar yield increased about 20.3% and 6.6% compared with that of the raw coal, respectively. Different with the traditional catalytic pyrolysis process for which the tar yield decreased, more tar was produced by the catalytic depolymerization method. The GC × GC-MS analysis showed that the components of alkanes and two-ring aromatics in tar increased dramatically with the addition of the optimal CoCl_2_ and ZnCl_2_ catalyst. The Raman measurement revealed that more large aromatic ring systems ( > 6 rings) were formed in the char from depolymerization with catalyst and the elemental analysis showed that the hydrogen content decreased slightly. The gas products analysis reflected the contents of methane showed an obvious decrease. All of this reflects that more fragments and small free radicals cracked were enhanced by catalysis in the catalytic depolymerization process, thereby leading to an enhancement of the tar formation possibilities.

## Introduction

Yunnan lignite contributing 12.5% of the total lignite reserves in China (estimated at more than 17 billion tons), which is considered as an ideal material for extracting its potential oil and gas resources for it contains high volatiles. Pyrolysis is perceived as an effective method which provides mild conversion of lignite into value added liquid fuels i.e. tar. However, in conventional coal pyrolysis process, the tar yield as well as the quality are handicapped by the low ratio of hydrogen to carbon of coal^1^. Catalytic pyrolysis is one of the most attractive techniques for increasing the coal conversion and optimizing the tar quality at mild conditions, which can be achieved by either blending the catalyst directly with coal or arranging the catalyst separately with coal to catalytic cracking of the volatiles^2^. Zou *et al*.^3^ impregnated 10 wt% of metal chlorides (CaCl_2_, CoCl_2_, NiCl_2_, KCl and ZnCl_2_) in Huolinhe lignite and found that CaCl_2_, ZnCl_2_ inhibited the conversion of organic matters into light species, while KCl, NiCl_2_ and CoCl_2_ promoted the lignite conversion. Oztas *et al*.^4^ blended 5~20 wt% metal chlorides with Zonguldak bituminous coal and found that CoCl_2_ and ZnCl_2_ increased the coal conversion. Han *et al*.^5^ also found that the 10 wt% of CoCl_2_ and ZnCl_2_ impregnated char had a good catalytic effect on the cracking of the volatiles, the yield of light tar fraction increased significantly. Then it could be concluded that catalytic pyrolysis of coal could promote the coal conversion or increase the specific components^2–6^. Moreover, CoCl_2_ and ZnCl_2_ were shown to be active catalysts for coal conversion and tar upgrading. However, in these studies, loading a large amount of catalyst were all needed, which would raise a host of problems such as high production cost, serious corrosion equipment and the difficult recovery of the catalyst. The failing of solving these problems greatly restricted the industrial application of the catalytic pyrolysis process.

Over the last 70 years, scientists have generated a large number of molecular level representations of coal^7^. Accordingly, the macromolecular structure of the coal is consists of hydroaromatic and aromatic condensed clusters linked by some basic bonds with different dissociation energies^7,8^. For the pyrolysis process, if the added catalysts can adhere into the macromolecular structure of the coal and cleave some bonds with higher energies selectively and directional control the stabilization of the hydrogen free radicals, the liquid products yield could be increased and the optimum product distributions would be possibly obtained. Consequently, a novel idea of catalytic depolymerization was proposed. Different from conventional catalyst addition method where the catalyst mainly distribute on the outside surface of coal, with the accessory ingredient assistance an intimate contact between the coal matrix structure and catalyst could be achieved^9^. It is expected that the catalytic activity would be enhanced and the amount of the catalyst could be decreased greatly by this method.

In this paper, based on the previous researchers’ study, CoCl_2_ and ZnCl_2_ were taken as basic catalysts. With the assistance of the accessory ingredient^10^, the effect of concentrations of the CoCl_2_ and ZnCl_2_ on the catalytic depolymerization of Yunnan lignite was studied. The optimal concentrations of the catalysts that could increase the tar yield was obtained. In order to achieve a better understanding of the catalytic depolymerization of Yunnan lignite, the product distribution at optimal conditions were analyzed and the possible reasons on tar yield increases were discussed.

## Results and Discussion

### Effect of concentrations of catalysts on tar yield

**Figure 1:**
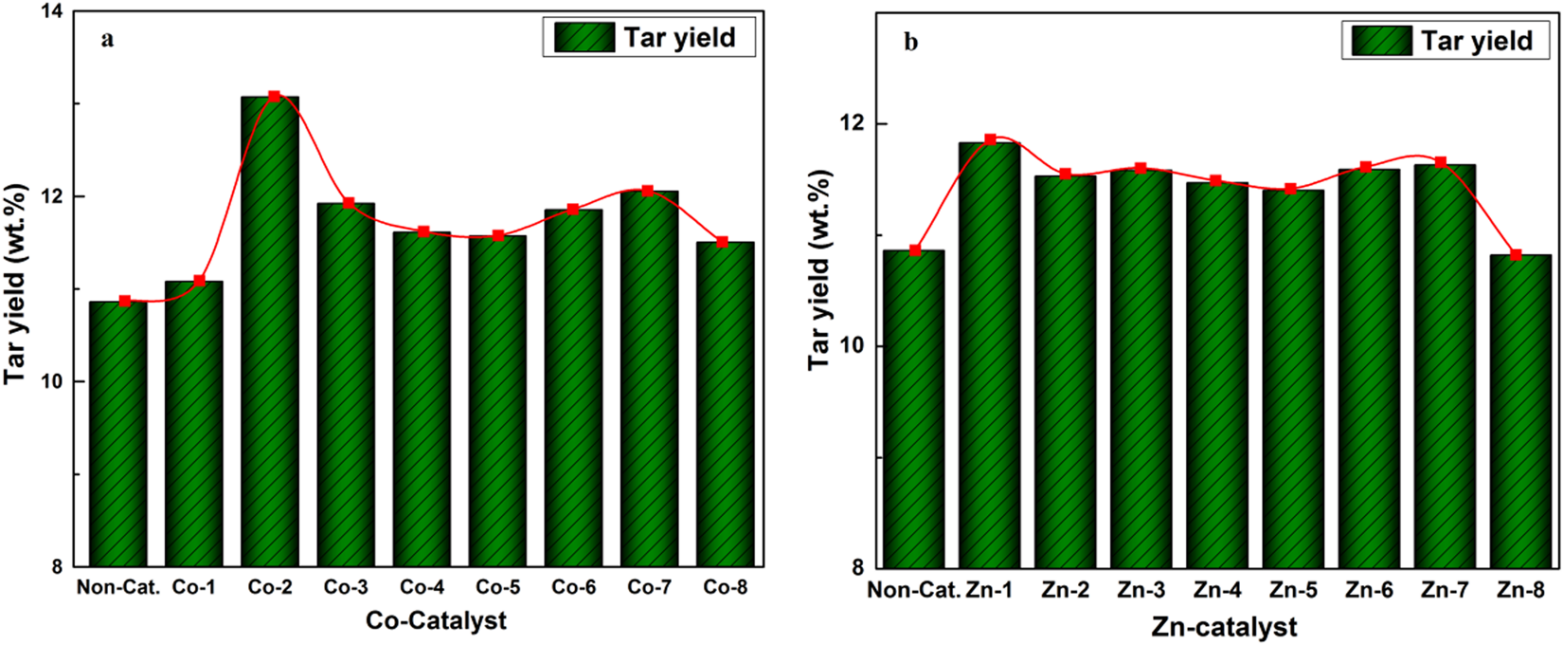
The effect of the concentrations of catalysts on tar yield.

Figure 1 presents the effect of loading amount of CoCl_2_ and ZnCl_2_ catalyst on tar yield (dry basis). As shown in Fig. 1a, compared with the tar yield of 10.86 wt.% for the raw coal decomposition, the tar yield increased initially with the increasing additive amount of CoCl_2_ catalyst, and the highest tar yield was obtained by the addition of Co-2 catalyst, for which the CoCl_2_ concentration was of 1.84 × 10^−3^ gram per gram of coal (dry basis) and the obtained tar yield was 13.07 wt.%. However, the increasing trend of the tar yield exhibited a rapid decrease and a minor fluctuations when the amount of the CoCl_2_ was higher than that of Co-2. Similar tendency was also observed with addition of ZnCl_2_ catalyst (Fig. 1b). The tar yield increased from 10.86 wt.% to its maximal value of 11.58 wt.% when Zn-1 catalyst was added (the ZnCl_2_ concentration is of 0.50 × 10^−3^ gram per gram of coal, dry basis). Compared with Fig. 1a and b, the highest tar yields were all obtained at relatively low concentrations of CoCl_2_ and ZnCl_2_ catalyst. Lower catalyst loading amount might be beneficial to catalyst dispersion and minimalize the secondary decomposition of tar, whereas excessive catalyst loading amount might contribute to aggregation of catalyst^11^, which would reduce the catalytic activity.

**Figure 2:**
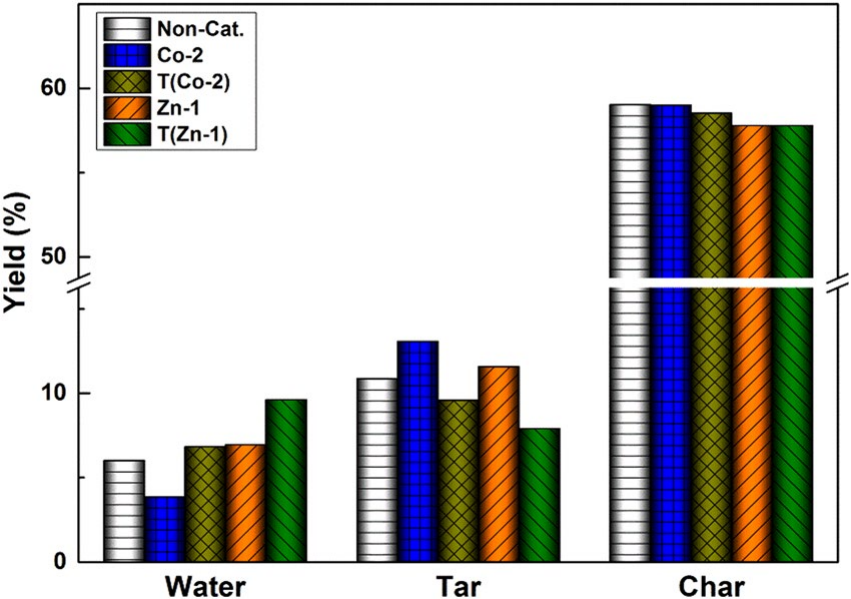
The products distribution with addition of catalyst Co-2 and Zn-1 by different methods.

In order to further clarify the difference of the catalytic depolymerization method with the traditional catalytic pyrolysis method, at the optimal catalyst concentrations of Co-2 and Zn-1, two groups of contrast experiments were designed. The same loading amount of catalyst (denoted as T(Co-2) and T(Zn-1)) was dissolved into distilled water and then impregnated to coal particles, and the operating conditions of the experiment were the same as the catalytic depolymerization method. As depicted in Fig. 2, compared with the T(Co-2) and T(Zn-1), the char yield did not change evidently by the catalytic depolymerization method, but the tar and water yield showed a big difference, indicating that the catalytic depolymerization might have experienced a different reaction process. Accordingly, the traditional catalytic pyrolysis mainly catalytic the reactions of the volatiles^2–6^, thus the tar yield decreased. For example, for T(Co-2), the tar yield decreased by 11.7%, while for Co-2, the tar yield increased by 20.3% differently.

### Tar fraction and composition analyses

Table 1 lists the variation of the tar fractions of the depolymerization of YN lignite with or without catalyst. Compared with the tar fractions that without catalyst, adopting catalysts significantly decreased the fractions of the light oil, phenol oil and anthracene oil. While the fractions of the naphthalene oil and wash oil, which mainly contained two-ring aromatics, showed an obvious increase, indicating that more chemical bonds related to this fraction temperature ranges (230–300 °C) were cleaved. The above results suggested that the addition of the two efficient catalysts not only increased the tar yield but also altered the composition of the tar.

**Table 1:** The amount of the tar fractions of the depolymerization of YN lignite with or without catalyst.

Component (%)	Temperature range (°C)	Non-Cat. tar	Co-2 tar	Zn-1 tar
Light oil	110–170	17.61	5.66	6.44
Phenol oil	170–210	19.28	14.69	16.67
Naphthalene oil	210–230	10.44	11.01	14.25
Wash oil	230–300	34.32	42.05	47.62
Anthracene oil	300–360	13.66	12.34	9.88
Pitch	>360	4.68	14.26	5.14

**Figure 3:**
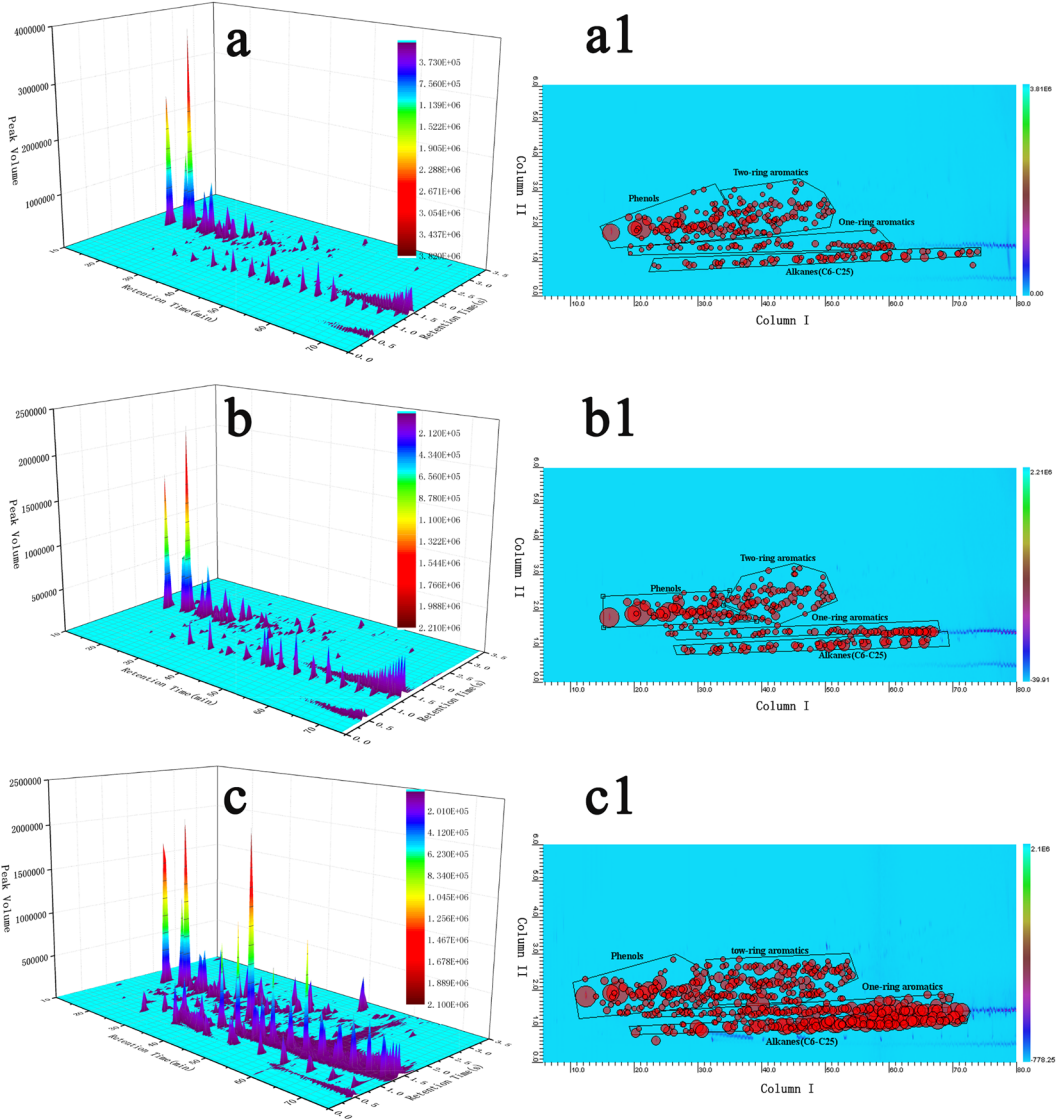
GC × GC-MS chromatogram of the different tar. 3D contour plot (left), and 2D contour plot (right): **a**, **a1**, tar from raw coal; **b**, **b1**, Co-2 tar; **c**, **c1**, Zn-1 tar. The marked zones on the 2D contour plot represented phenols, one-ring aromatics, two-ring aromatics and alkanes.

**Figure 4:**
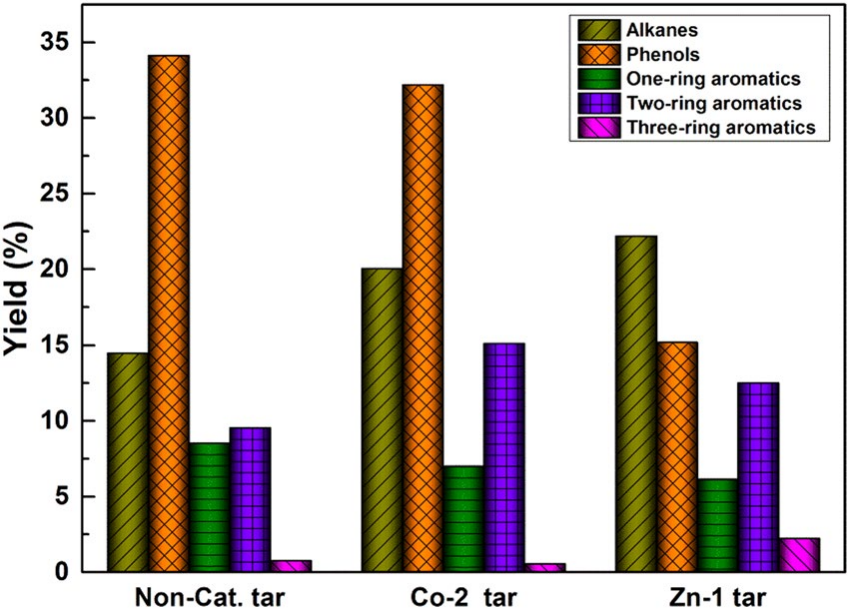
The variation of composition of tar with and without catalyst.

For a better comparison of the tar components, two dimensional gas chromatography coupled with mass spectroscopy (GC × GC-MS) were used^12,13^. Figure 3 illustrates the three dimensional (3D) and two dimensional (2D) contour plot of the tars from the depolymerization of raw coal with and without catalyst. For the 3D contour plot, the X-axis of GC × GC chromatogram is the volatility-based retention time (min), the Y-axis is the polarity-based retention time (s), and the Z-axis is the MS response. Based on the NIST library and peak volume, the proportions of the detected components in the liquid products were determined. The 2D contour plot are the top view of the 3D contour plot and the bubble plot of all peak apices represent the different species in the tar. From Fig. 3 it could be seen that GC × GC-MS has a very high interfamily resolution and the tar components are well separated. Moreover, the tar components could be categorized into four regions of alkanes, phenols, one-ring aromatics, two-ring aromatics by the two-dimensional retention time. Figure 4 further clarifies the tar composition varying with the addition of two catalysts. Compared with the tar composition from the raw coal pyrolysis, the contents of phenols decreased by 5.7% and 55% in the presence of catalyst Co-2 and Zn-1, respectively, and the contents of one-ring aromatics also decreased. On the contrary, the contents of two-ring aromatics (mainly naphthalene) and alkanes (mainly C7-C25) increased obviously. For example, the addition of Co-2 catalyst increased the contents of two-ring aromatics and alkanes by 57.3% and 38.5%, respectvely. Besides, the addition of Zn-1 catalyst increases the contents of three-ring aromatics. These results were consistent with the tar fraction analysis. The addition of the two optimal catalysts makes the coal depolymerized into heavier components.

### Gas composition analysis

**Figure 5:**
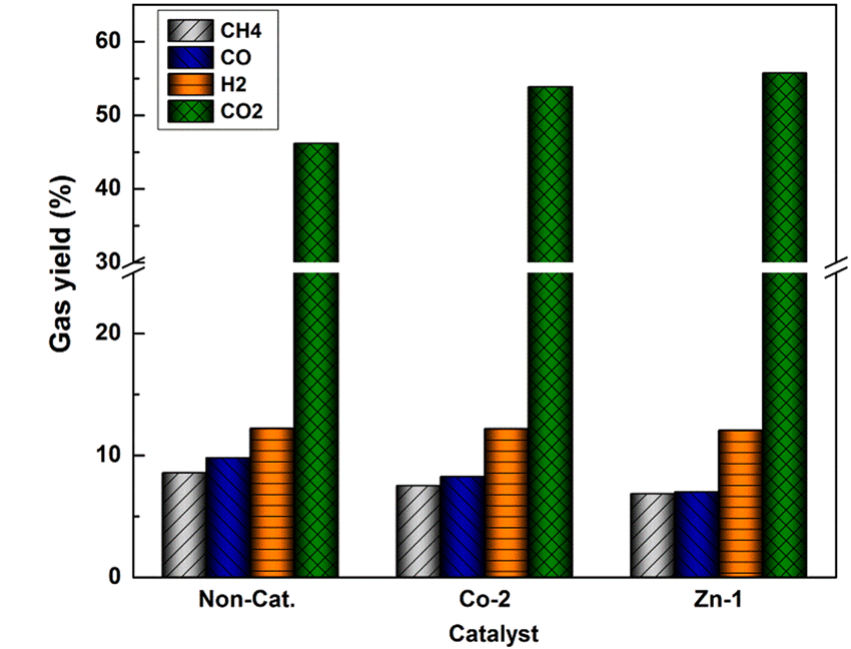
The gas composition varying with addition of the catalysts.

Figure 5 exhibits the effect of the Co-2 and Zn-1 catalyst on the gas composition. The carbon monoxide and methane contents showed a slight reduction, while the contents of carbon dioxide increased dramatically compared with that of raw coal. The contents of hydrogen almost remained constant with the adding the catalysts. The tar formation reactions follow a radical mechanism, beginning with the breakdown of covalent bonds to form radical fragments and followed by coupling of those fragments to generate tar^14^. The extra small radicals such as CH_x_^∙^ and H^∙^ can either react with each other to form into small molecular gases or react with some radical fragments to form macromolecule tar. Consequently, the decrease of the methane content might partially be responsible for the tar increases, for which would provide some more intermediates CH_x_ and H^∙^.

### Char characterization

Table 2 shows the elemental analysis of the residual chars with and without catalysts. For the chars from the depolymerization of coal with Co-2 and Zn-1 catalyst, the hydrogen content decreased slightly from 2.46% to 2.35% and 2.38%, respectively, while the carbon contents all increased. This suggests that the catalysts might promote the transformation of hydrogen to other phases and enhance the condensation between char fragments, so as the condensation degree of the chars with catalyst increased.

**Table 2:** Ultimate analyses of chars with and without catalysts.

	C_d_	H_d_	N_d_	S_t,d_	H/C(mol/mol)
Non-Cat. char	66.10	2.46	1.61	0.99	0.45
Co-2 char	66.29	2.35	1.52	0.99	0.43
Zn-1 char	66.70	2.38	1.61	0.98	0.43

_d_, dry; _t,d_, total sulfur dry basis.

**Figure 6:**
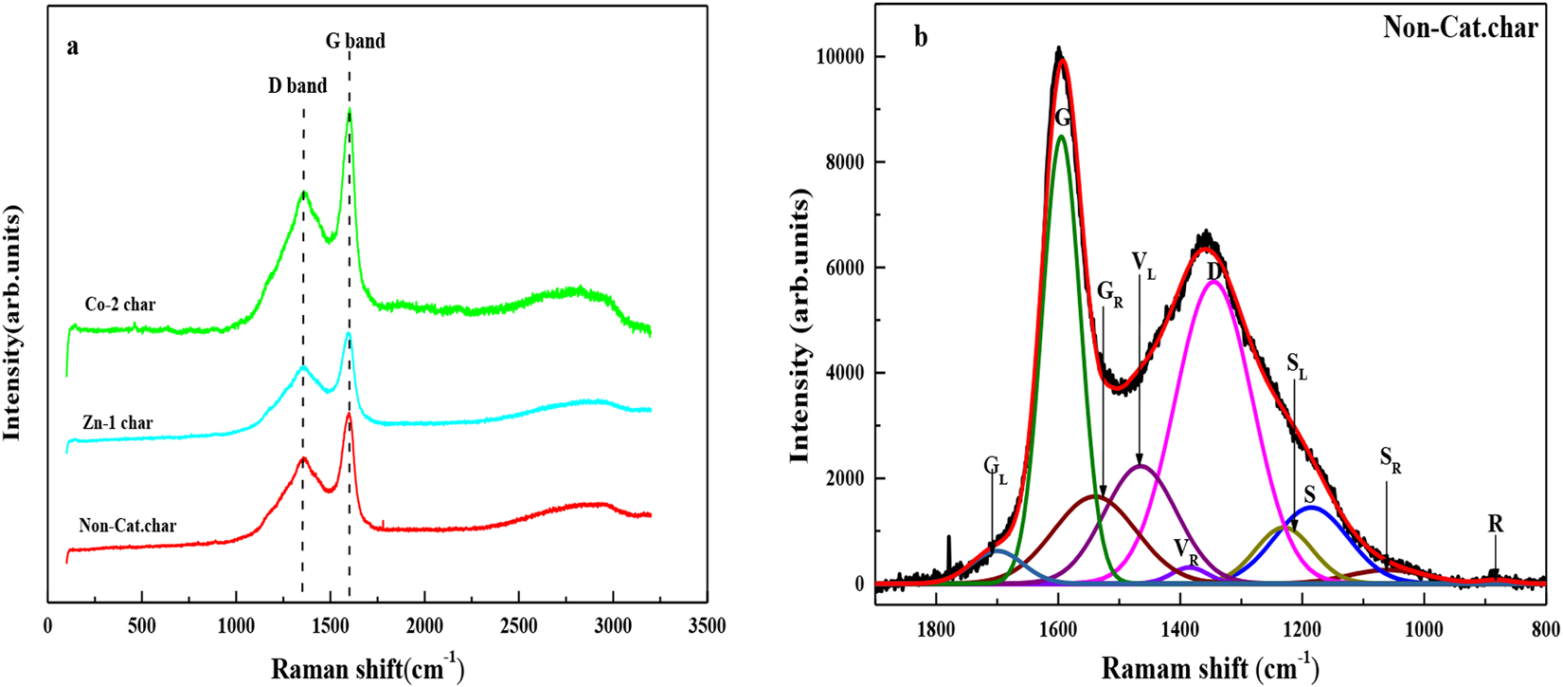
(**a**) Raman spectra of char samples; (**b**) Raman spectra of char without catalyst fitted 10 bonds.

Raman spectroscopy analysis was performed in order to improve the understanding of the microcrystalline structure of the residual char samples. Figure 6a illustrates the Raman spectra of the different chars. All the curves exhibited two broad and overlapping peaks with maximal intensity at 1340–1380 cm^−1^ and 1580–1600 cm^−1^, which correspond to the D bond and G bond, respectively. The Raman spectra of chars in the range between 800 and 1800 cm^−1^ represent the major typical structures in the chars, which could be curve-fitted into 10 Gaussian bonds^15^. An example of spectral curve-fitting of a Raman spectrum of the raw char is shown in Fig. 6b. As seen, peaks G, G_R_, V_L_, V_R_, D and S were the six main bands to reflect structural information of the coal char^15^. In this approach, A_D_ is the area of the D band mainly representing the large aromatic ring systems with no less than 6 fused rings. A_G_ is the area of the G band mainly representing the disordered graphite structure in coal. A_(GR+VL+VR)_ is the total area of the (G_R_ + V_L_ + V_R_) bands, representing typical structures in amorphous carbon, mainly due to small aromatic ring systems (less than 6 fused rings). The A_D_/A_G_ ratio represents the degree of aromatic ring growth. The A_D_/A_(GR+VR+VL)_ was considered as a brief proportion of larger (≥6 fused rings) aromatics to the smaller (3–5 fused rings) aromatics in the chars.

As illustrated in Table 3, the A_D_/A_G_ and A_D_/A_(GR+VL+VR)_ ratios of chars in the presence of catalysts were all increased compared with the char without catalysts. The decrease of the relative ratio of the smaller aromatics reflected the increase of the condensation degree of the chars. It also revealed that the smaller aromatic ring systems are preferentially activated and transformed with the addition of catalyst, which resulted by the dehydrogenation of hydroaromatics^15^. From the gas composition analysis, the contents of hydrogen in the gases did not increased. While the contents of two-ring aromatics (mainly naphthalene) and alkanes (mainly C7-C25) in the tar increased obviously. One reason might be the addition of the catalyst might promote the cracking of the bonds with higher energies, such as the smaller aromatics which was bonded with the macromolecular structure of the coal. Another reason might be the catalyst activate the molecular hydrogen originated from char condensation, inducing bonds cleavage and stabilizing radical fragments to form tar^16^.

**Table 3:** The two parameters deduced from Raman spectroscopy for char samples.

Sample	A_D_/A_G_	A_D_/A_(GR+VL+VR)_
Non.-Cat. char	1.38	1.44
Co-2 char	1.43	1.53
Zn-1 char	1.46	1.62

### Possible reasons on the increasing of the tar yield

Coal structure mainly consists of a series of covalent bonds e.g. C_ar_-C_ar_, C_ar_-C_al_, C_ar_-H, C_al_-C_al_, C_al_-H, C_al_-O, -S-^7,8^. The breakdown of these bonds depends on the bonds’ energies and pyrolysis temperature^17^. During pyrolysis process, the bonds with lower energy (C_al_-C_al,_ C_al_-O, -S-, C_al_-H) would be broken with the temperature increase, while the bonds with higher energies (C_ar_-C_ar_, C_ar_-C_al_, C_ar_-H) are hard to be broken. For the traditional catalytic pyrolysis, the catalyst is added and it’s believed that the catalyst would stay outside of the coal. It might only affect the volatiles reformation, as Fig. 2 shows, the tar yield would decreases for the secondary decomposition reactions. Different with that, using our method, the catalyst was added to coal with the assistance of the accessory ingredient^10^, for which its’ hoping that the catalyst could be added into the coal macromolecular structure, then the catalyst could interact with the coal structure and directional promote the cracking of the bonds with higher dissociation energies and finally increases the tar yield^9^.

In the experiment, the highest tar yield were all obtained at relatively low concentrations of CoCl_2_ and ZnCl_2_ catalyst, and the further increasing amount of CoCl_2_ and ZnCl_2_ catalyst did not increase the tar yields, reflecting the lower catalyst loading amount might be beneficial for the catalyst dispersion and minimalize the secondary decomposition of tar. It’s also found that CoCl_2_ and ZnCl_2_ catalyst shows different catalytic effect on Yunnan lignite. This might be caused by the difference of the electron cloud distribution of the CoCl_2_ and ZnCl_2_ catalyst, which may further effect the location and interaction of the catalyst with the coal macromolecular. If we use the increasing extent of the tar yield to evaluate the catalytic effect of the two catalysts, CoCl_2_ is better than ZnCl_2_.

**Figure 7:**
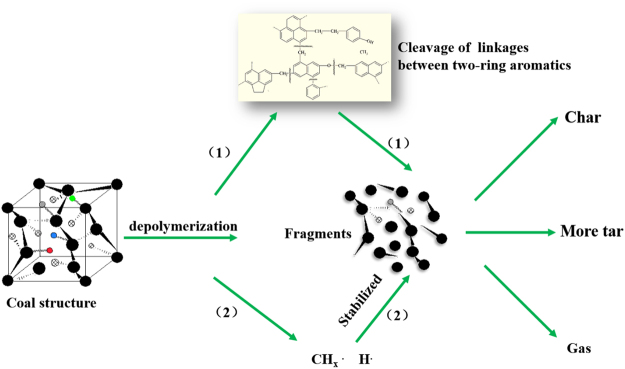
The possible route of catalytic depolymerization for increasing tar yield.

Moreover, the two optimal catalysts also showed similar catalytic effect on the products compositions such as the two- or three-ring aromatics and alkanes in tar increased obviously, the methane in gases decreased and the condensation degree of the chars increased. The catalytic effect of the two optimal catalysts essentially reflecting their special interactions with covalent bonds during depolymerization process, and the increasing of the tar yield might be deduced by the following process: With the assistance of the accessory ingredient, CoCl_2_ or ZnCl_2_ catalyst located on the macromolecular structure of the coal, which is consists of a series of covalent bonds. The catalyst interact with the macromolecular structure of the coal and catalyzed the cleavage of the linkages between aromatic unites during catalytic depolymerization process, generating more fragments. As presented in the route (1) of Fig. 7, more two- or three-ring aromatics fragments are generated. At the same time, as illustrated in the route (2) of Fig. 7, the small free radicals formed (CH_x_^.^ and H^.^) would be favored to combine with the extra two- or three-ring aromatics fragments, thus leading to an enhancement of tar formation possibilities.

## Conclusions

The catalytic depolymerization of Yunnan lignite over CoCl_2_ and ZnCl_2_ catalyst can improve the tar yield obviously. Compared with the raw coal, at very low catalyst loading amount the tar yield increases by 20.3% (Co-2 catalyst, for which the CoCl_2_ concentration is of 1.84 × 10^−3^ gram per gram of coal) and 6.6% (Zn-1 catalyst, for which the ZnCl_2_ concentration is of 0.50 × 10^−3^ gram per gram of coal). Meanwhile, the components of alkanes and two-ring aromatics in tar are also enhanced. Combined with the analyses of the gases and char, it is concluded that the two optimal catalysts used can promote the breakdown of linkages connected structural units of coal, especially for the linkages between smaller aromatic units, leading to more fragments generation which were further stabilized by the additional small free radicals (CH_x_^.^ and H^.^), and finally responsible for tar increase. It is proved that the method of adding catalyst into coal in this work has an improving effect on the tar yield.

## Materials and Methods

### Coal and catalyst preparation

**Figure 8:**
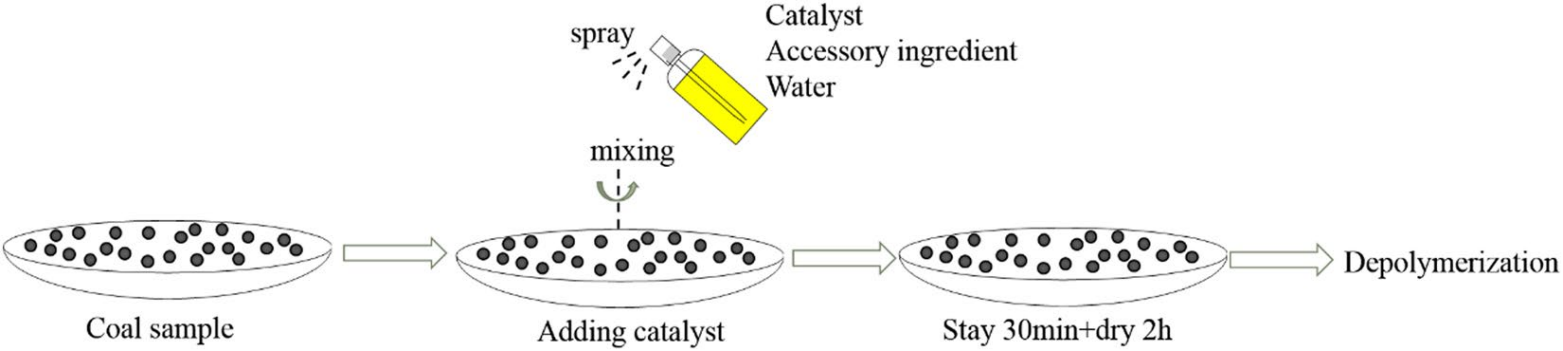
The process of catalyst loading.

The applied coal sample was Yunnan lignite (YN) that obtained from Yunnan province, China. The sample was pulverized to pass through a 80-mesh sieve (particle size less than 0.178mm) for use. The proximate and ultimate analyses of the sample were listed in Table 4. The catalyst addition procedure was shown in Fig. 8. Metal chloride of ZnCl_2_/CoCl_2_·6H_2_O was dissolved into the distilled water with specific additives at room temperature^10^, and then the solutions were sprayed into 30 g of coal (air dried basis) evenly followed by intensive mixing. After stayed for 30 min, the treated coal was dried in a vacuum oven at 105 °C for 2 h and then cooled down to room temperature. As the catalyst was dissolved into the distilled water, the loading concentration of CoCl_2_·6H_2_O catalyst was calculated by CoCl_2_ amount and the catalyst was recorded as CoCl_2_ for simplification. The loading amount of the catalyst in per gram of dried coal was presented in Table 5. The catalyst that contains 0.91 × 10^−3^ gram of CoCl_2_ catalyst per gram of coal is denoted as Co-1. The catalyst that contains 0.50 × 10^−3^ gram of ZnCl_2_ catalyst per gram of coal is denoted as Zn-1.

**Table 4:** Proximate and ultimate analyses of YN lignite.

Proximate analysis (wt.%)			Ultimate analysis (wt.%),d				
M_ad_	A_d_	V_daf_	C	H	O^*^	N	S_t_
7.64	12.81	56.66	57.24	4.42	23.62	1.13	0.78

_ad_, air dry; _d_ dry; _daf_, dry ash free; ^*^, by difference; ^t^, total content.

**Table 5:** The loading amount of the catalyst.

No.	CoCl_2_ amount (×10^–3^ g/g)	No.	ZnCl_2_ amount (×10^–3^ g/g)
Co-1	0.91	Zn-1	0.50
Co-2	1.84	Zn-2	1.00
Co-3	3.69	Zn-3	2.00
Co-4	7.37	Zn-4	4.00
Co-5	11.06	Zn-5	6.00
Co-6	18.43	Zn-6	8.00
Co-7	27.64	Zn-7	10.00
Co-8	44.00	Zn-8	15.00

### Apparatus and procedures

The experiments of the coal depolymerization with and without catalysts were performed by a Gray-King apparatus^18^. The experiments were tested in a horizontal electrically-heated quartz tube with a length of 300 mm and inner diameter of 20 mm. The procedure of the tests was set according to the Chinese standard of GB/T 1341–2007. About 10 g of the prepared coal samples were placed in the quartz tube evenly, then it was heated with a heating rate of 5 °C/min to the final temperature (600 °C) and was kept at this temperature for 15 min. The liquid products involving tar and water were collected in a conical flask with an ice-salt bath. Then the water in the liquid products was separated according to the Chinese standard of GB/T480–2010, using toluene as a solvent. In this way, tar and water yield can be calculated respectively. The gas was collected and analyzed by gas chromatography (Haixin GC-920) with a TCD detector. All the experiments were repeated three times and the standard deviation of the product yields were found to be less than 0.5%.

### Tar fractions analysis

Tar was analyzed via a Setaram SETSYS TGA^9^. About 10 mg of tar were placed in a alumina crucible and then heated to 360 °C with a heating rate of 10 °C/min at a argon flow rate of 100 mL/min. The tar fractions were divided into six parts by temperature ranges: light oil (<170 °C), phenol oil (170–210 °C), naphthalene oil (210–230 °C), washing oil (230–300 °C), anthracene oil (300–360 °C) and pitch (>360 °C).

### Characterization of tar with GC × GC-MS

In this paper, the hydrocarbon components of the tar were analyzed by Agilent GC × GC-MS, which composed of an Agilent GC7890B system equipped with an Agilent 5977 A MSD mass spectrometer, and a ZX1 thermal modulator (Zoex) was connected in the GC oven to achieve a three-dimensional separation. The initial oven temperature was set to 70 °C, and then ramped to 300 °C at a heating rate of 10 °C/min. The mass range scanned was 30–500 m/z. The volume of sample injected was 1 uL using a spilt ratio of 50:1. The carrier gas was Helium (99.999%). The compounds were identified by comparing mass spectra with NIST08 and NIST08s library data.

### Raman spectroscopy analysis

Raman spectra for the study of the structure defects of the coal char samples were performed at room temperature using a RENISHAW in Via Raman Microscope with laser power 20 mW × 5% (1 mV), the laser of excitation wavelength of 514.5 nm was chosen. The Raman spectra were recorded in the range between 800 and 1800 cm^−1^.

## Electronic supplementary material

Supplementary material
