# Supplementary material for: Catalytic depolymerization of a typical lignite for improving tar yield by Co and Zn catalyst
Source: Sci Rep. 2017 Oct 31;7:14433. doi: 10.1038/s41598-017-14869-w (PMC5663918; doi:10.1038/s41598-017-14869-w)
Supplement: Supplementary file 1 — Supplementary material [file 41598_2017_14869_MOESM1_ESM.doc]

**SUPPLEMENTARY MATERIAL**

**Catalytic depolymerization of a typical lignite for improving tar yield by Co and Zn catalyst**

Litong Liang^1^, Juntian Huai^2^, Qian Zhang^1^, Jianwei Liu^1^, Wei Huang^1,*^, Zhonglin Zhang ^3^, Xiaogang Hao^3^, Guoqing Guan^4^

^1^ *Key Laboratory of Coal Science and Technology of Ministry of Education and Shanxi Province, Taiyuan University of Technology, Taiyuan 030024,* *Shanxi, China*

^2^ *State Key Laboratory of Coal-based Low carbon Energy, ENN Group Co., Ltd, China*

^3^ *Department of Chemical Engineering, Taiyuan University of Technology, Taiyuan 030024, Shanxi, China*

^4^ *North Japan Research Institute for Sustainable Energy, Hirosaki University, 2-1-3 Matsubara, Aomori 030-0813, Japan*

*^*^ Corresponding author. Tel: (+86) 0351 6018073; E-mail address:* [*huangwei@tyut.edu.cn*](mailto:huangwei@tyut.edu.cn)


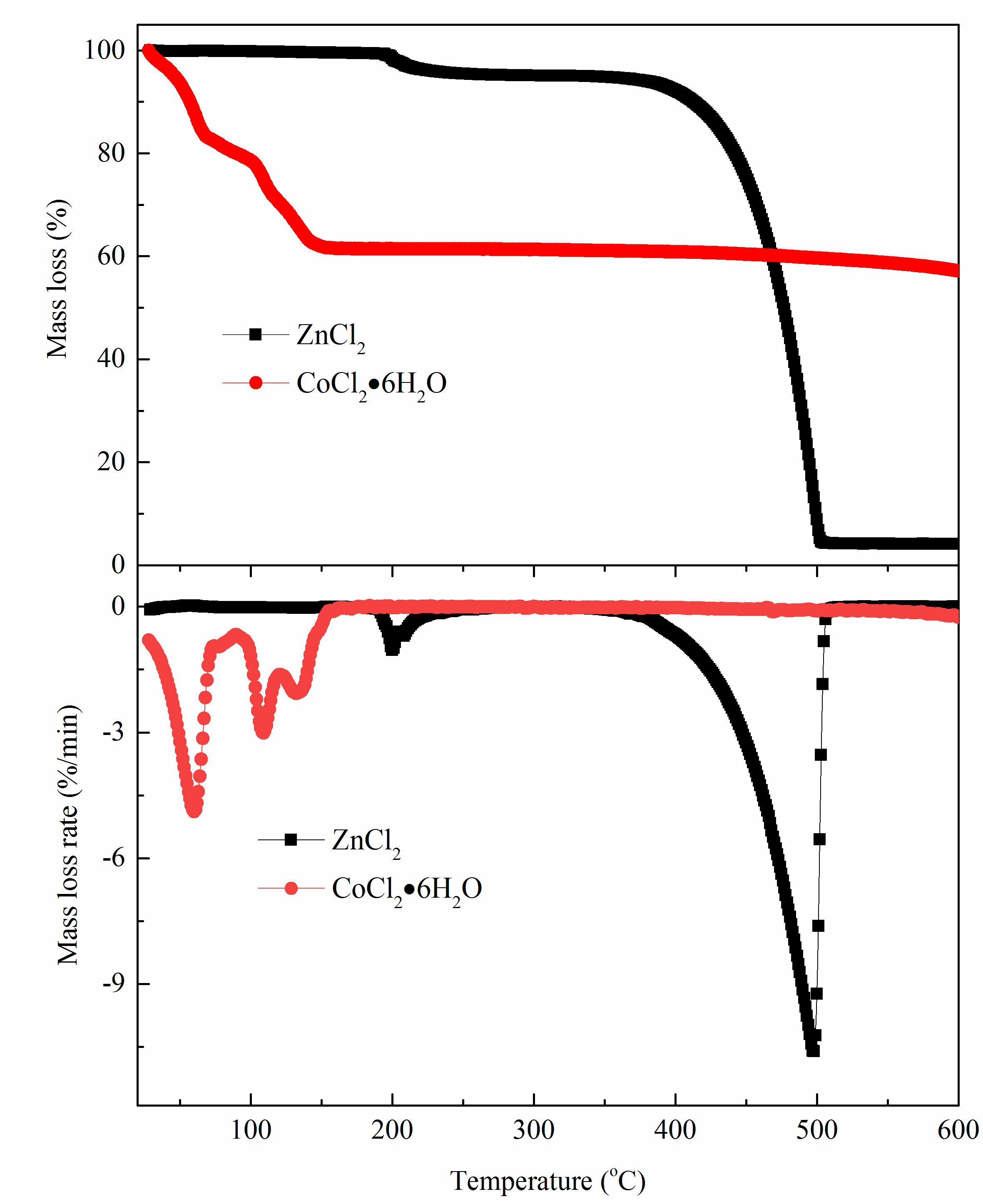


Fig. S1. TG and DTG results of ZnCl_2_ and CoCl_2_·6H_2_O

Fig. S1 shows the thermal depolymerization behavior of the ZnCl_2_ and CoCl_2_·6H_2_O catalyst with a heating rate of 5 ^o^C/min at an argon flow rate of 100 mL/min. The CoCl_2_·6H_2_O shows an obvious mass loss at the temperature range of 25-150 ^o^C, which is mainly caused by the escape of combined water (about 40 wt.%), and after that, little mass loss is appeared. Theoretically, the content of the combined water in CoCl_2_·6H_2_O is about 45 wt.%, while some of the combined water might evaporated as CoCl_2_·6H_2_O is exposed to air at room temperature before the test, so the mass loss is about 40 wt.% at the temperature range of 25-150 ^o^C in the experiment. For ZnCl_2_, there is a slight mass loss (about 3 wt.%) appeared at the temperature of 200 ^o^C, which might be caused by the decomposition of the combined water or impurities. After that, when the temperature is higher than 400 ^o^C, though this temperature is still does not come to the boiling point of ZnCl2 (732 ^o^C), it evaporated obviously.

In this paper, the thermal depolymerization behavior of the ZnCl_2_ and CoCl_2_ catalyst was not considered for the adding content of the catalyst is very low, especially for the optimal catalyst, the Co-2 and Zn-1, for which the CoCl_2_ and ZnCl_2_ catalyst content in the coal is about1.84×10^-3^ and 0.50×10^-3^ gram per gram of coal (dry basis), respectively. The evaporation effect of the catalyst on the product yield is very limited.
